# Evaluating the Role of PTH in Promotion of Chondrosarcoma Cell Proliferation and Invasion by Inhibiting Primary Cilia Expression

**DOI:** 10.3390/ijms151119816

**Published:** 2014-10-31

**Authors:** Wei Xiang, Ting Jiang, Fengjing Guo, Tao Xu, Chen Gong, Peng Cheng, Libo Zhao, Weiting Cheng, Kai Xu

**Affiliations:** 1 Department of Orthopedics, Tongji Hospital, Tongji Medical College, Huazhong University of Science and Technology, Wuhan 430030, China; E-Mails: xiangwei_13@163.com (W.X.); fengjing_guo@126.com (F.G.); pengcheng201507@163.com (P.C.); zhaolibotj@163.com (L.Z.); 2 Department of Rehabilitation, Tongji Hospital, Tongji Medical College, Huazhong University of Science and Technology, Wuhan 430030, China; E-Mails: jiangting0601@163.com (T.J.); xutao0101@yeah.net (T.X.); 3 Department of Oncology, Tongji Hospital, Tongji Medical College, Huazhong University of Science and Technology, Wuhan 430030, China; E-Mail: gongchengonco@126.com; 4 Department of Oncology, Wuhan Integrated Traditional Chinese Medicine and Western Medicine Hospital, Wuhan No1. Hospital, Wuhan 430030, China; E-Mail: weitingcheng@yeah.net

**Keywords:** chondrosarcoma, primary cilia expression, parathyroid hormone-related protein, PTHrP

## Abstract

Chondrosarcoma is characterized by secretion of a cartilage-like matrix, with high proliferation ability and metastatic potential. Previous studies have shown that parathyroid hormone-related protein (PTHrP) has a close relationship with various tumor types. The objectives of this study were to research the function played by PTHrP in human chondrosarcoma, especially targeting cell proliferation and invasion, and to search for the potential interaction between PTHrP and primary cilia in tumorigenesis. Surgical resection tissues and the human chondrosarcoma cell line SW1353 were used in the scientific research. Cells were stimulated with an optimum concentration of recombinant PTH (1-84), and siRNA was used to interfere with internal PTHrP. Cell proliferation and invasion assays were applied, including MTS-8 cell proliferation assay, Western blot, RT-PCR, Transwell invasion assay, and immunohistochemistry and immunofluorescence assays. A high level of PTHrP expression was found in human chondrosarcoma tissues, and recombinant PTH exhibited positive promotion in tumor cell proliferation and invasion. In the meantime, PTHrP could inhibit the assembly of primary cilia and regulate downstream gene expression. These findings indicate that PTHrP can regulate tumor cell proliferation and invasion ability, possibly through suppression of primary cilia assembly. Thus, restricting PTHrP over-expression is a feasible potential therapeutic method for chondrosarcoma.

## 1. Introduction

Chondrosarcoma, as a type of tumor producing a cartilage-like matrix, is one of the most common primary malignant bone tumors. This tumor generally occurs in patients aged more than 20 years [1,2,3], and the 10-year survival rate ranges from 29% to 83%, based on the tumor’s degree of differentiation [4,5]. Because of its lack of vascularity and its high expression of a cartilage-like matrix, chondrosarcoma has been confirmed to be less sensitive to routine chemotherapy and radiation therapy. Surgical resection is still considered the best approach in treating chondrosarcoma [6]. Therefore, the study of the pathogenesis mechanism of chondrosarcoma has great significance in guiding the selection of treatment methods and developing novel therapies.

Parathyroid hormone-related protein (PTHrP) is a crucial factor in regulating calcium balance in the internal environment, and it plays an indispensable role in the processes of bone and cartilage growth and differentiation [7,8,9,10,11]. In normal cartilage, PTHrP can inhibit cartilage cell differentiation and promote proliferation by a PTHrP/Ihh feedback loop [12]. PTHrP was found to be regulated by the expression of Hedgehog (Hh) signaling pathway downstream transcription zinc finger protein GLI2 and to suppress the expression of effective Ihh protein in turn [7,10]. This feedback loop can control cartilage cells’ maturation and postpone the process of endochondral ossification, thus helping to maintain bone structure and function [12].

However, a high level of PTHrP may not only lead to teratogenesis, but it also may induce malignancies, such as giant cell tumor of bone and various bone metastasis tumors. These tumors were detected in high expression of PTHrP that leads to metastatic osteolytic destruction accompanied with hypercalcemia [8,9,10,11]. In the current study, we confirmed that human chondrosarcoma tissues expressed a high level of PTHrP, compared with adjacent tissues, and this protein contributes to positive facilitation in promoting proliferation and invasion. What is more, this auxoaction realization is through the regulation of Hh signaling pathway-associated primary cilia disassembly.

Primary cilia, as a type of extracellular organelle, have multifarious functions. For example, primary cilia can serve as a control center for many signaling pathways, such as Hh and Wnt molecules [13,14,15,16]. They also can detect extracellular mechanical stress stimulation and biochemical environment changes [17,18,19]. In addition, primary cilia assembly is a sign of the end-point of the cell division cycle [20]. Also, intraflagellar transport protein IFT88, activated mainly in primary cilia, represents the function of internal material transport in cilia [21]. Previous studies have demonstrated that deficiency of primary cilia has a close link with tumorigenesis, such as breast cancer, gastrointestinal tumor, renal carcinoma, and others [16,22,23,24].

Therefore, through studying the interaction between PTHrP and primary cilia, we have been able to initiate a new way to research the pathogenesis mechanism of chondrosarcoma and provide a foothold for guiding clinical treatment of chondrosarcoma with great practical significance.

## 2. Results and Discussion

### 2.1. Human Chondrosarcoma Tissue Expressed an Elevated Level of PTHrP

**Figure 1:**
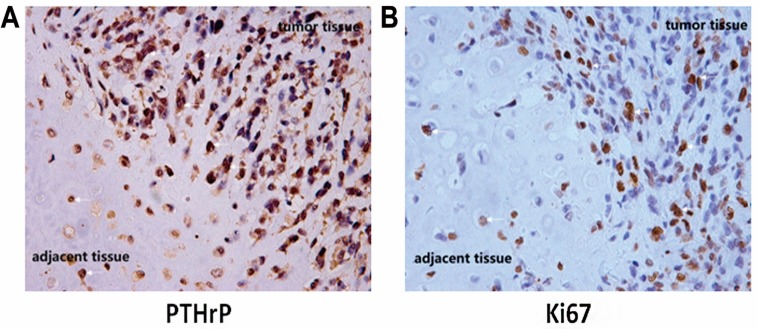
Human chondrosarcoma tissues expressed elevated level of parathyroid hormone-related protein (PTHrP) accompanied by strong proliferation ability. The white arrows refer to positive tumor cells. The left image (**A**) shows PTHrP expression in chondrosarcoma tumor tissue and adjacent tissue; The right image (**B**) shows the Ki67 expression.

PTHrP protein plays a crucial role in the process of normal bone tissue development [7,8,9,10,11,12]. Abnormal expression of PTHrP has been found in various tumor types. In this study, through immunohistochemical staining, we found that human chondrosarcoma tissues apparently expressed an elevated level of PTHrP, compared with adjacent tissues (Figure 1). Meanwhile, nearly half of the total tumor cells (44.30% ± 4.52%) were stained positive with Ki67 primary antibody, which was considered a biomarker for proliferation. Previous study confirmed that an Ihh/PTHrP negative loop exists in normal cartilage growth and the differentiation process. PTHrP contributed to keeping cartilage cells in the proliferation stage, rather than differentiation [12]. These results confirmed that the unusual expression of PTHrP may be essential for promoting chondrosarcoma cell proliferation.

The left image shows that chondrosarcoma tumor tissue expressed apparent PTHrP greater than adjacent tissue. Nearly half of the tumor cells were stained positively, compared to paracarcinoma tissue (right image). We collected five surgically resected specimens of chondrosarcoma in immunohistochemical staining. 

### 2.2. PTH Promotes Chondrosarcoma Cell Proliferation by Suppressing Primary Cilia Assembly

**Figure 2:**
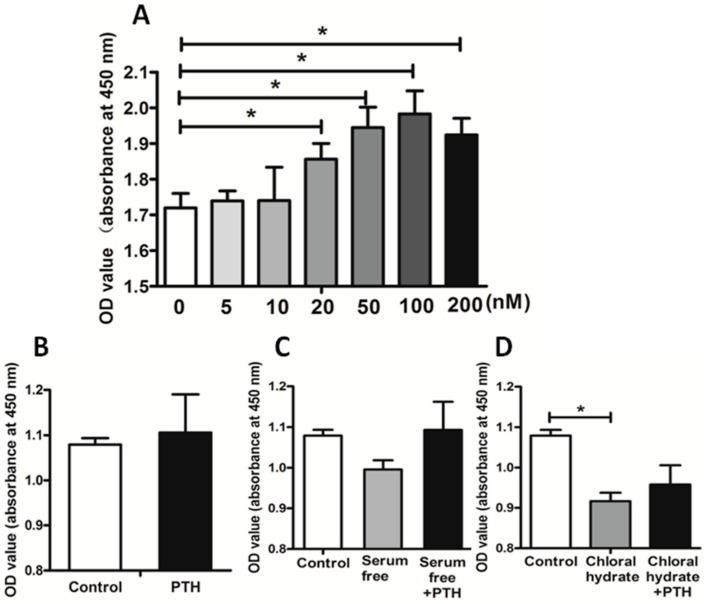
PTH promoted SW1353 cell proliferation by acting on primary cilia. Image **A** shows the effect of different concentrations of PTH on SW1353 proliferation; Image **B** shows the effect of PTH on tumor cell; Image **C** shows the effects that serum free medium and PTH on tumor cell proliferation; Image **D** shows the influences that chloral hydrate and PTH stimulation on proliferation ability. (* *p* < 0.05).

PTH is believed to be a crucial factor in regulating tumor growth. It always leads to bone destruction with consequent hypercalcaemia due to tumor cells’ proliferation and invasion [10]. In order to study the mechanism of PTH effects on tumor cell proliferation, we treated chondrosarcoma cells with different doses of recombinant PTH (1-84). The results indicated that the facilitation effect increased serially in a range, with 100 nM the optimal concentration to promote proliferation (Figure 2A).

Subsequently, we added 100 nM PTH to both the serum-free culture medium and the chloral hydrate culture medium. We observed that PTH could significantly reverse cell proliferation inhibition through a serum-free medium, which could cause cells to go into a stationary phase and induce primary cilia assembly. Further, PTH had little positive effect on the chloral hydrate-treated tumor cells (Figure 2D). Chloral hydrate was thought to be a high-efficiency component to destroy the basal body structure of primary cilia [16]. Therefore, we speculated that PTH may facilitate chondrosarcoma cell proliferation by interacting with primary cilia. By destroying the primary structure of cilia, the up-regulated proliferation ability caused by PTH was restrained significantly.

SW1353 cells were treated with different concentrations of recombinant PTH (1-84), and there is a trend that cell proliferation increased and reached the peak at 100 nM (Figure 2A). A serum-free medium was used to induce primary cilia assembly, and chloral hydrate (40 μM) to destroy them. Adding PTH (100 nM) could improve cilia assembly cell proliferation ability, rather than perturbing cilia cells (Figure 2B–D). We used a microplate reader to assess the proliferation by the absorbance at 450 nm wavelength. Three independent experiments were conducted (* *p* < 0.05, *n* = 5).

### 2.3. PTH Enhances Chondrosarcoma Cells Invasion Capacity by Interacting with Primary Cilia

**Figure 3:**
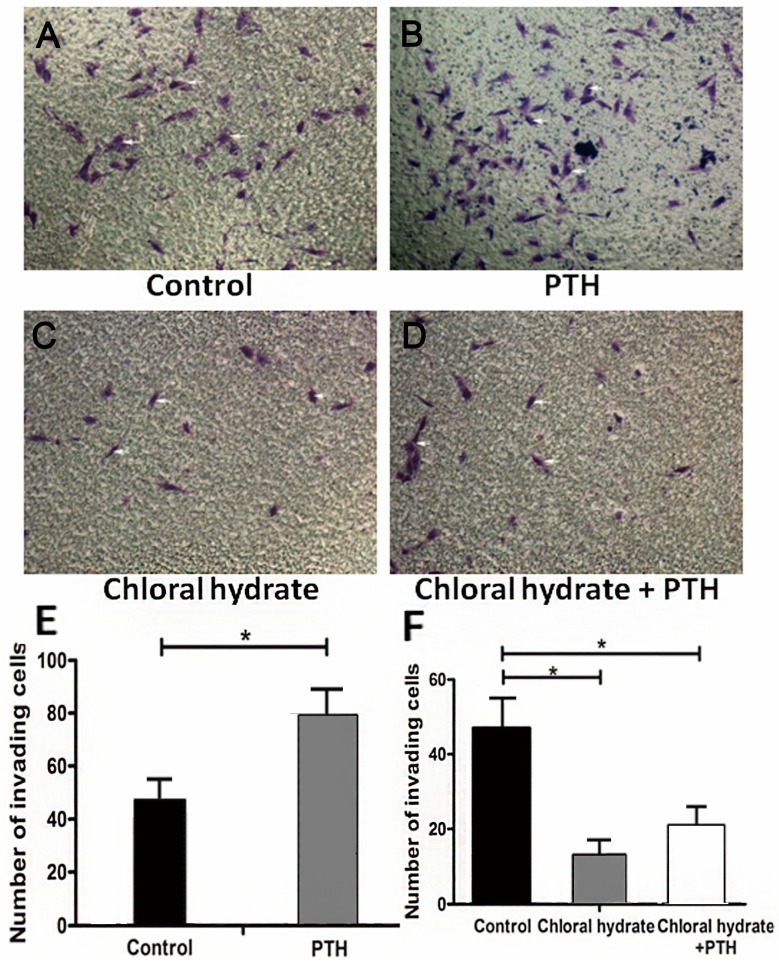
PTH enhances chondrosarcoma cells’capacity for invasion by interacting with primary cilia. The white arrows refer to tumor cells. Image **A** is the control group; Image **B** shows PTH stimulation on tumor cell invasion ability; Image **C** shows the effect that chloral hydrate stimulation on tumor cell invasion; Image **D** shows the influence that PTH caused after stimulating tumor cell on chloral hydrate; These histograms (**E** and **F**) display the average number of invading cell on different conditions. (* *p* < 0.05).

The destructive force of tumor cells in decomposing the extracellular matrix reflects the invasion ability of tumor cells. Our Transwell invasion assays showed that more PTH-treated cells penetrated to the lower surface than in the empty control group (Figure 3A,B, *p* < 0.05). After we used chloral hydrate to destroy the cilia structure, these transmembrane cells decreased and could not make a reversion by being treated with PTH (Figure 3A,C, *p* > 0.05). This phenomenon illustrated that primary cilia could play a crucial role in PTH-mediated promotion of tumor cells penetrating the extracellular matrix process. 

Transwell assays were used to assess tumor cells’invasion ability. As these photos show, PTH (100 nM) could significantly facilitate the cells’ penetration to the lower surface (*p* < 0.05), while PTH could not improve invasion ability after destroying cilia with chloral hydrate (*p* < 0.05) (Figure 3A). This histogram displays the average number of invading cells (B). Three independent experiments were conducted. A *p*-value less than 0.05 was defined as a statistically significant criterion.

**Figure 4:**
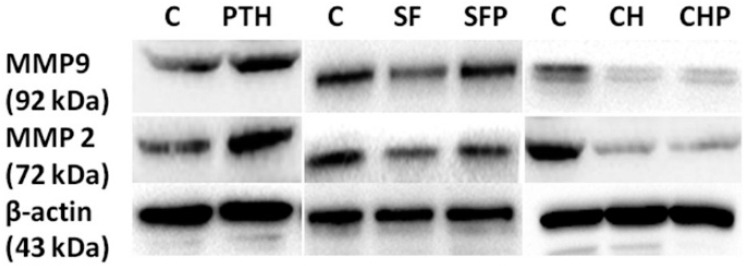
PTH could regulate MMP2 and MMP9 proteins expression by primary cilia.

Western blot assays (Figure 4) also demonstrated that simple PTH could up-regulate expression of tumor cell invasion-associated matrix metalloproteinases MMP2 and MMP9, especially post-treated with serum-free starvation. But after destroying primary cilia with chloral hydrate, MMP2 and MMP9 were down-regulated and irrelevant in the presence of PTH. These results revealed that PTH could facilitate chondrosarcoma cells’invasion ability through interaction with primary cilia to initiate relevant function. Suffering from external damage to primary cilia had a negative regulatory effect for invasion.

PTH (100 nM) (P) could increase MMP2 and MMP9 expression to some extent, and improve serum-free (SF) medium-treated cells’ MMP expression. However, after using chloral hydrate (CH) to destroy cilia, PTH could not affect the expression of both proteins. Three independent assays were conducted.

### 2.4. PTH/PTHrP Can Regulate Primary Cilia Expression

**Figure 5:**
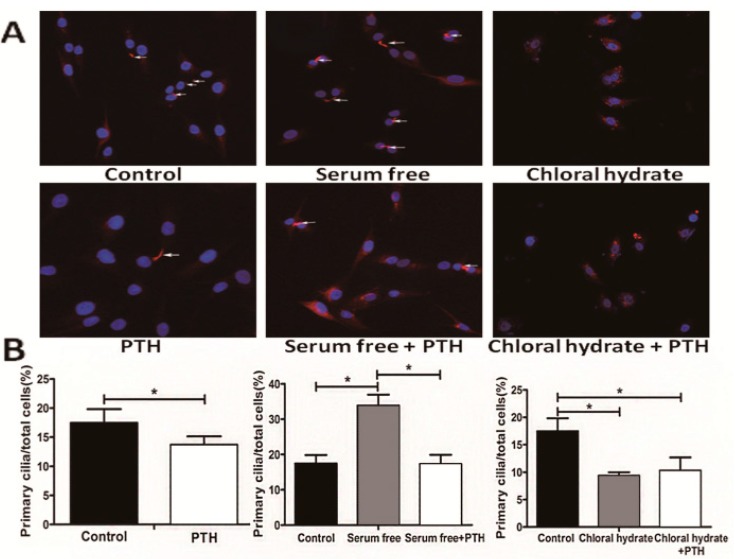
PTH could regulate SW1353 cells primary cilia assembly. These white arrows refer to primary cilia. Image A shows primary cilia expression on different condition and these histograms (B) display the percentage of primary cilia expression. (* *p* < 0.05).

**Figure 6:**
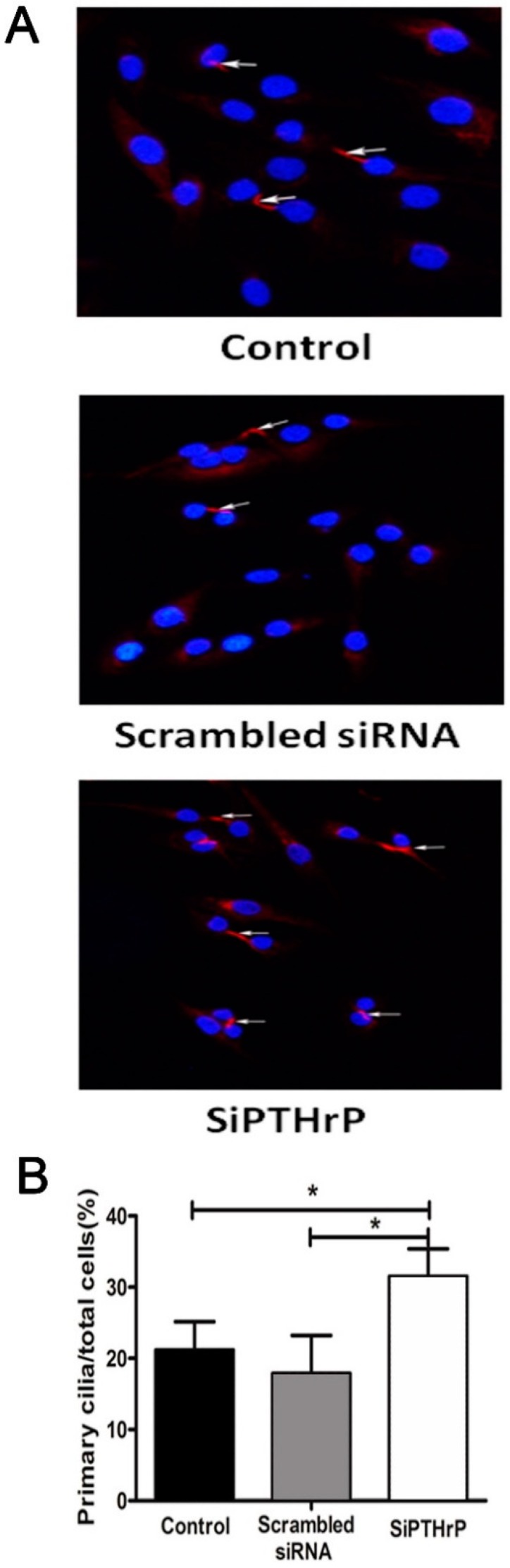
PTHrP silence could increase primary cilia expression. (A) Using siRNA to silence PTHrP gene expression caused elevated ciliogenesis, compared with the empty control (*p* = 0.003) and negative (*p* = 0.000) groups; (B) The relative percentage of primary cilia in different treatment groups is shown. Three independent experiments were conducted. The white arrows refer to primary cilia. (* *p* < 0.05).

PTH, as an important endocrine-regulation factor, is a downstream effect molecule of Hh pathway. Primary cilia are a crucial part of the classical Hh pathway upstream structure in controlling cell cycle course [20]. We conjectured theoretically that growth factor PTH may regulate the cells’ growth progression by affecting primary cilia. In order to validate this hypothesis, we observed primary cilia from a morphologic view (Figure 5 and Figure 6). We first induced ciliogenesis with a serum-free culture medium and then added PTH to stimulate chondrosarcoma cells. Newly appearing primary cilia could be suppressed significantly by PTH (33.88% ± 3.12% to 17.35% ± 2.54%, *p* < 0.001).

Primary cilia were stained with acetylated α-tubulin and identified by morphologic characteristics; positive acetylated α-tubulin (red) staining. Nuclei are stained with 4',6-diamidino-2-phenylindole (DAPI) (blue). (A) Different treatment groups of chondrosarcoma cells displaying primary cilia are indicated by white arrows. PTH could down-regulate cilia expression, whether in a normal medium (*p* < 0.05) or a serum-free medium (*p* < 0.001 but has less effect on chloral hydrate-stimulated cells (*p* = 0.554). (B) Comparison of the percentage of different treated cells presenting primary cilia in chondrosarcoma is shown. At least three independent experiments were conducted.

At the same time, if we used chloral hydrate to destroy primary cilia and then treated them with PTH, primary cilia showed no significant difference (9.38% ± 0.62% to 10.33% ± 2.67%, *p* > 0.05). What is more, when we used chemical synthetic small interfering RNA to down-regulate endogenous PTHrP expression, the percentage of primary cilia rose distinctly, compared with the empty control (31.50% ± 3.90% to 21.18% ± 3.97%, *p* < 0.05) and negative control (31.50% ± 3.90% to 17.88% ± 5.32%, *p* < 0.001) groups (Figure 6).

**Figure 7:**
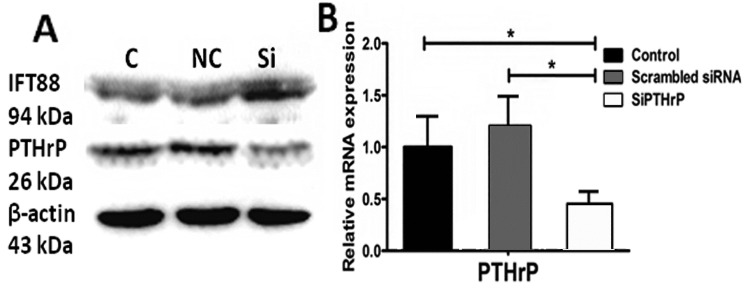
siRNA could decrease PTHrP expression in both protein and gene levels. The left image A shows the effect of siRNA on PTHrP and IFT88 proteins expression; The right one (B) display the interfere efficiency of siRNA on PTHrP in gene level. (* *p* < 0.05).

Furthermore, knocking down endogenous PTHrP was accompanied by slightly elevated expression of cilia relevant intraflagellar transport protein IFT88 (Figure 7). These outcomes reminded us that PTH/PTHrP may have a reverse-regulation impact on primary cilia morphology expression and eventually achieve function depending on complete cilia.

We used small interference RNA to reduce PTHrP RNA; three days later, PTHrP protein and gene expression decreased distinctly, compared with the empty control (*p* < 0.05) and negative control (*p* < 0.05) groups. At the same time, reducing PTHrP could slightly elevate cilia-related intraflagellar transport protein IFT88 expression. Three independent experiments were conducted.

### 2.5. PTH/PTHrP Can Affect Hh Signaling Pathway Downstream Gene Expression

**Figure 8:**
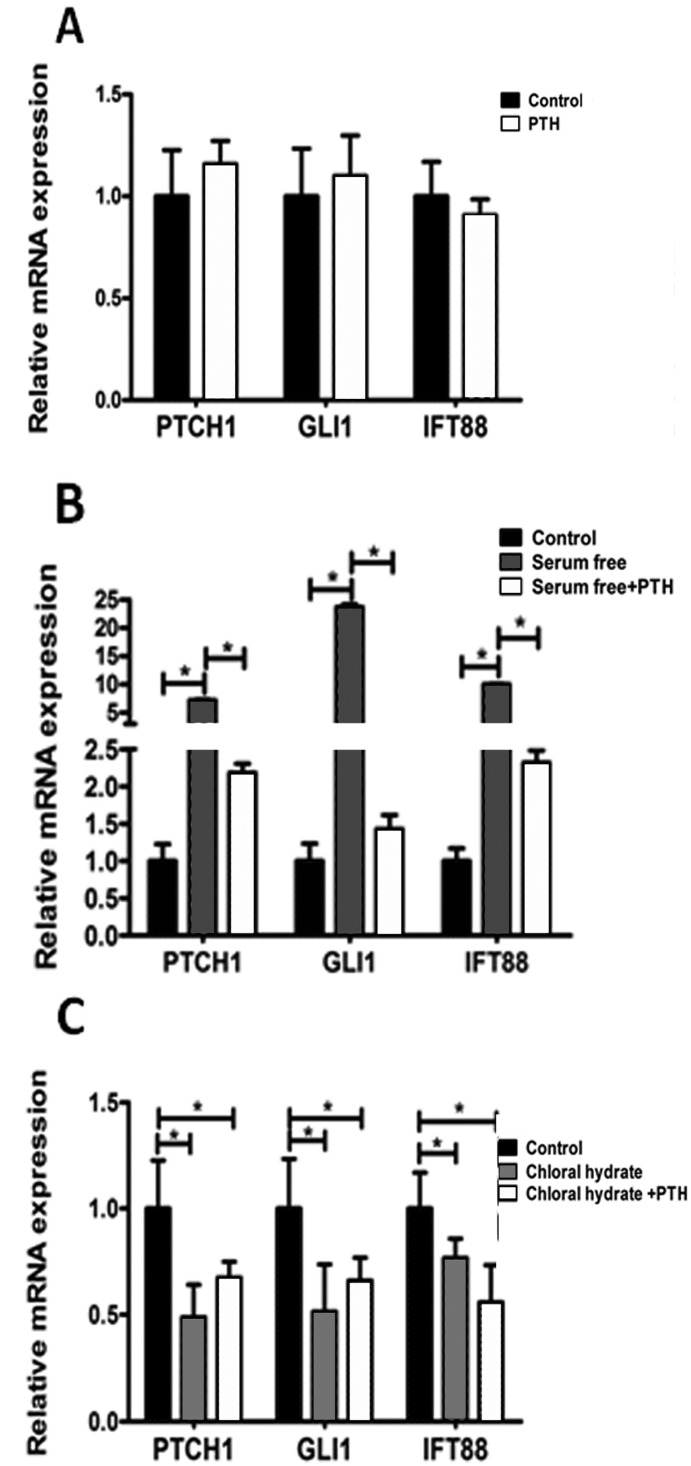
PTH/PTHrP can affect Hh signaling pathway downstream gene expression. (**A**) Treatment with recombinant PTH (10 nM) alone did not alter the expression of Hh pathway-related genes PTCH1, GLI1, and IFT88 significantly (*p* > 0.05); (**B**) After inducing cilia by serum-free medium, Hh pathway was activated and PTH could suppress target genes expression significantly (*p* < 0.001); (**C**) Treatment with chloral hydrate down-regulated target genes to a certain extent, and PTH could not distinctively change the trend (*p* > 0.05). Three independent assays were conducted. (* *p* < 0.05).

As a vital component of Hh signaling pathway, primary cilia play an important role in the transferring signal [16]. Intraflagellar transport protein 88 (IFT88) is the most important transporting signal molecule in primary cilia [21]. In addition, GLI1 and PTCH1 have been generally recognized as the target genes of Hh signaling pathway [16]. We used RT-PCR to detect the influences that PTH/PTHrP imposed on Hh signaling pathway. As Figure 8 shows, these results suggested that simple treatment with PTH had no obvious effect on Hh pathways, compared with the control group (Figure 8A, *p* > 0.05), while the pathways could be activated by serum-free stimulated and increased IFT88, GLI1, and PTCH1 genes’expression, to some extent (Figure 8B, *p* < 0.05). In addition, PTH could down-regulate Hh signaling pathway (Figure 8B, *p* < 0.001) by inhibiting ciliogenesis. After destroying primary cilia by chloral hydrate, PTH had no significant effects on downstream gene expression (Figure 8C, *p* > 0.05). 

In addition, Western blot assay showed that in reducing endogenous PTHrP, intraflagellar transport protein IFT88 expression was somewhat higher (Figure 7A). These findings illustrated that PTH/PTHrP could affect Hh downstream gene expression through a negative regulation of primary cilia.

As a key regulatory factor, PTHrP plays an important part in the growth and development of bone and cartilage tissue *in vivo*. In normal cartilage, PTHrP secretes through autocrine and paracrine matter. Binding with PTHrP receptor-1, PTHrP can delay chondrocyte differentiation and promote cell proliferation to regulate the developmental processes of cartilage [7,8,9,10,11,12]. However, abnormal expression of PTHrP can cause a range of metabolic function disorders *in vivo* [8]. There have been a variety of studies that found malignancy accompanied with high expression of PTHrP is also associated with hypercalcemia [7,8,9,10,11]. PTHrP can activate oncogene Bcl-2 expression and is considered to be an upstream regulator of Bcl-2 [25]. Otherwise, PTHrP is also regarded as a major regulator of bone tumor-caused osteolytic destruction [8,9,10,11]. These studies suggested that the abnormal expression of PTHrP has a close relationship with the occurrence of malignancy.

As a special type of tumor that can secrete cartilage matrix, chondrosarcoma occurs from abnormal development of cartilage. The level of malignancy depends on the degree of tumor cell differentiation [16]. Although the specific pathogenesis is not clear, in this study we found that human chondrosarcoma tissues had a distinct PTHrP expression, and the expression levels were positively correlated with the tumor proliferation rate.

This phenomenon suggests that aberrant expression of PTHrP may cause cell proliferation to be increased while differentiation is inhibited, leading to the occurrence of chondrosarcoma. Various studies have shown that PTH is the decisive factor that causes osteolytic destruction in primary or metastatic bone tumors [8,9,10,11]. In our *in vitro* experiments, an appropriate concentration of PTH (1-84) could promote proliferation of SW1353 human chondrosarcoma cells and strengthen the ability to secrete more matrix metalloproteinase MMP2 and MMP9, thereby enhancing degradation of extracellular matrix and facilitating the ability to migrate. The Transwell invasion assay results also confirmed this conclusion. Thus, PTHrP is likely to play a key role in regulating chondrosarcoma dedifferentiation during development.

Primary cilia, as cell superficial structures, can detect the changes of extracellular mechanical stimulation and biochemical environment [17,18,19]. Primary cilia are considered to be a critical point of many signaling pathways, regulating interactions. Previous studies have found that normal cartilage cells express a higher level of primary cilia than neoplastic chondrocytes [16], by which they can feel external mechanical stress and internal osmotic pressure changes, thereby regulating cartilage cells’ growth and differentiation [17,19,26]. Primary cilia historically have been regarded as a biomarker for the end of division and out of cell cycle, which can objectively reflect the cell proliferation stage, and the inner intraflagellar transport protein IFT88 represents the physiologic function of cilia [20,21].

## 3. Experimental Section

The study was approved by the Ethics Committee of Tongji Medical College, Huazhong University of Science and Technology, Wuhan, Hubei, China.

### 3.1. Cells and Reagents

The human chondrosarcoma cell line SW1353 was purchased from the Type Culture Collection of the Chinese Academy of Sciences, Shanghai, China. Cells were cultured in Dulbecco’s modified Eagle medium/nutrient mixture F-12 (DMEM/F-12) with 10% fetal bovine serum, penicillin (100 U/mL) and streptomycin (100 U/mL) and incubated at 37 °C with 5% CO_2_. Human recombinant PTH (1-84) was purchased from ProSpec, Ness-Ziona, Israel.

### 3.2. Cell Proliferation Test

We used an assay of 3-(4,5-dimethylthiazol-2-yl)-5-(3-carboxymethoxyphenyl)-2-(4-sulfophenyl)-2*H*-tetrazolium (MTS-8) to evaluate the cell proliferation rate. The steps were as follows: 2000 cells per well were plated in 96-well plates. Different stimulation reagents were added to the cells based on the experiment’s design. We added 100 μL DMEM/F12 with 10% fetal bovine serum and incubated for a suitable time. Then 10 μL MST-8 (Boster, Wuhan, China) was added to each well. Two hours later, the OD value (absor-bance at 450 nm wavelength) was measured using an enzyme micro-plate reader. The cell viability was expressed by the OD value. 

### 3.3. Transwell Invasion Assay

Transwell assay was used to assess chondrosarcoma cells’ invasion ability. The upper filter membrane (pore diameter, 8 μm) of the Transwell plates was coated with 25 mg Matrigel (BD Biosciences, San Jose, CA, USA) at 37 °C environment for 30 min. The lower chambers were filled with cell culture medium DMEM/F-12 containing 10% fetal bovine serum. SW1353 cells were first starved in serum-free DMEM for 4 h. After digestion 1 × 10^5^ cells were transferred onto the upper surface. Based on experimental design, we added either 100 nM PTH, or 40 μM chloral hydrate and also both. Chloral hydrate was used to disrupt the junction between primary cilium and the basal body. Twenty-four hours later, we gently wiped the upper surface of the Matrigel. Paraformaldehyde at 4% was used to fix the cells and allowed to penetrate to the lower surface for 15 min. After washing three times, we stained the cells with crystal violet (Boster, Wuhan, China). Ten random visual fields were selected and counted under the micro-scope (Olympus, Tokyo, Japan).

### 3.4. Immunohistochemical Studies

Human chondrosarcoma tissues were fixed in 4% paraformaldehyde. We then embedded tissues in paraffin and sectioned it for immunohistochemical assays. All these experimental processes were conducted using standard techniques based on previous studies [9,16]. Primary antibodies PTHrP (1:100 dilution, Santa Cruz Biotechnology, Santa Cruz, CA, USA) and Ki67 (1:200 dilution, Cell Signaling Technology, Danvers, MA, USA) were stained. All the sections were observed, and photos were taken under microscopic magnification ×200.

### 3.5. Immunofluorescence Assay

Chondrosarcoma SW1353 cells of the proper density were inoculated on cover glasses and stimulated with different reagents. Twenty-four hours later, they were fixed with 4% paraformaldehyde for 15 min, blocked with 0.5% BSA at room temperature for 60 minutes, and then incubated with primary acetylated α-tubulin antibody (1:300 dilution, Abcam, Cambridge, UK) overnight at 4 °C. Subsequently, cells were incubated with CY3-conjugated goat anti-mouse IgG secondary antibody at room temperature, and the nuclei were stained with 1 µg/µL DAPI. PBS was used to wash the cells three times for 10 min during each step in this process. Finally, images were visualized and recorded with a fluorescent microscope.

### 3.6. Western Blot Analysis

The steps for Western blot analysis were as previously described [10]. Total cell proteins (40 μg/lane) were loaded and separated by use of sodium dodecyl sulfate (SDS)-polyacrylamide gels, and then the proteins were trans-ferred onto PVDF membranes. We incubated these membranes overnight with primary antibodies-MMP2 (1:1000 dilution, Cell Signaling Technology, Danvers, MA, USA); MMP9 (1:1000 dilution, Cell Signaling Technology, Danvers, MA, USA); IFT88 (1:500 dilution, ABGENT, San Diego, CA, USA); PTHrP (1:100 dilution, Santa Cruz Biotechnology, Santa Cruz, CA, USA); β-actin (1:400 dilution, Boster, Wuhan, China)-following up with secondary antibody horse-radish peroxidase-labeled goat anti-rabbit and goat anti-mouse (1:5000 dilution, Boster, Wuhan, China) IgG for one hour. The ECL Western blotting detection kit (Thermo Fisher Scientific, Geel, Antwerpen, Belgium) was used to detect all the protein bands and visu-alized using an enhanced chemiluminescence system (Bio-Rad, Philadelphia, PA, USA). All the values were expressed relative to β-actin.

### 3.7. Quantitative Real-Time PCR (qRT-PCR)

Ihh/PTHrP feedback loop-related genes (GLI1, PTCH1, IFT88, PTHrP) were measured by real-time PCR (RT-PCR). After incubation for 24 h with different stimulation reagents in six-well plates, RNA was extracted from the cells using RNeasy Mini Kit (Invitrogen Life Technologies, New York, NY, USA), according to the manufacturer’s instructions. cDNA was synthesized from 2 to 5 μg of total RNA using the First-Strand Synthesis System for RT-PCR (Invitrogen Life Technologies, New York, NY, USA), per the manufacturer’s instructions. The total PCR system contained cDNA, SYBR Green, no RNA enzyme, water and primers. The primer sequences were as outlined in the descriptions.

### 3.8. Small-Interfering RNA Transfection

SW1353 chondrosarcoma cells were transitorily transfected with 50 nM siRNA targeting PTHrP or with a scrambled sequence (Negative Control siRNA) for 6 h using Lipofectamine 2000 (Invitrogen Life Technologies, New York, NY, USA). Cells were maintained in growth media for 72 h. The efficiency of the knockdown specific gene was evaluated by qRT-PCR and Western blot assays, as previously described. Both small interfering RNA and negative control siRNA were synthesized by the Guangzhou RiboBio Company, Guangzhou, China.

### 3.9. Statistical Analysis

Each experiment was conducted at least three times. These data were represented as mean ± SD. We used the student’s *t*-test or one-way analysis of variance to analyze the differences among means. A *p*-value less than 0.05 was defined as statistically significant. All statistical analyses were performed using SPSS 20.0 (IBM, Armonk, NY, USA).

## 4. Conclusions

In this study, we found that recombinant PTH (1-84) can increase human chondrosarcoma cell proliferation activity, accompanied by inhibition of extracellular primary cilia generation and IFT88 expression. In addition, we used chloral hydrate to disrupt the junction of the cilium and the basal body to inactivate cilia. After destroying the cilia by means of chloral hydrate, treatment with recombinant PTH had no significant effect on Hh downstream genes GLI1 and PTCH1, together with intraflagellar transport protein IFT88. 

Interestingly, chemical destruction of the cilia may lead to the proliferation and invasion abilities distinctly inhibited in tumor cells. Therefore, we first noticed that through dependence on primary cilia, PTH can achieve its facilitation function and regulate ciliogenesis oppositely, eventually affecting the proliferation and invasion activity of tumor cells. Details of the specific mechanism of PTH acting on primary cilia are a possible focus of attention in future research.

The study of how PTHrP affects chondrosarcoma cell proliferation and invasion in regulating primary cilia expression have provided us with a new way of viewing the pathogenesis of chondrosarcoma. How to manipulate primary cilia assembly to restrain PTH-mediated tumorigenesis could be a question deserving in-depth investigation. These conclusions may have great significance in guiding future chemotherapy drug investigation and improving clinical treatment efficacy on human chondrosarcoma.
